# Recombinant *Lactococcus lactis* Carrying IL-4 and IL-10 Coding Vectors Protects against Type 1 Diabetes in NOD Mice and Attenuates Insulitis in the STZ-Induced Model

**DOI:** 10.1155/2021/6697319

**Published:** 2021-02-02

**Authors:** Tatiane M. Preisser, Vanessa P. da Cunha, Mariana P. Santana, Vanessa B. Pereira, Denise C. Cara, Bianca M. Souza, Anderson Miyoshi

**Affiliations:** ^1^Laboratory of Genetic Technology, Department of Genetics, Ecology and Evolution, Institute of Biological Sciences, Federal University of Minas Gerais, Belo Horizonte, Minas Gerais ZIP/Post Code: 31270-901, Brazil; ^2^Center for Gastrointestinal Biology, Department of Morphology, Institute of Biological Sciences, Federal University of Minas Gerais, Belo Horizonte, Minas Gerais ZIP/Post Code: 31270-901, Brazil

## Abstract

Type 1 diabetes (T1D) is an autoimmune disease that culminates in beta cell destruction in the pancreas and, subsequently, deficiency in insulin production. Cytokines play a crucial role in the development of diabetes, orchestrating the recruitment and action of immune cells, to not only destroy insulin-producing cells but also preserve them. Therefore, the aim of this study was to investigate the effect of orally administered *Lactococcus lactis* MG1363 FnBPA^+^ strains carrying plasmids encoding IL-4 and IL-10 in the streptozotocin- (STZ-) induced diabetes model and in nonobese diabetic (NOD) mice. The STZ-induced mice that were treated with combined bacterial strains carrying plasmids encoding IL-4 and IL-10 showed lower incidence of diabetes and more preserved pancreatic islets than the mice that received the individual bacterial strains. Combined administration of *L. lactis* MG1363 FnBPA^+^ (pValac::dts::*IL-4*) and *L. lactis* MG1363 FnBPA^+^ (pValac::*IL-10*) resulted in protection against diabetes in NOD mice. It was shown that the combined treatment with recombinant bacterial by oral route prevented hyperglycemia and reduced the pancreatic islets-destruction in NOD mice. In addition, increased levels of IL-4 and IL-10 in serum and pancreatic tissue revealed a systemic effect of the treatment and also favored an anti-inflammatory microenvironment. Reduced concentrations of IL-12 in pancreas were essential to the regulation of inflammation, resulting in no incidence of diabetes in treated NOD mice. Normal levels of intestinal sIgA after long-term treatment with the *L. lactis* strains carrying plasmids encoding IL-4 and IL-10 indicate the development of oral tolerance and corroborate the use of this potent tool of mucosal delivery. For the first time, *L. lactis* MG1363 FnBPA^+^ strains carrying eukaryotic expression vectors encoding IL-4 and IL-10 are tested in STZ-induced and NOD mouse models. Therefore, our study demonstrates this innovative strategy provides immunomodulatory potential for further investigations in T1D and other autoimmune diseases.

## 1. Introduction

Type 1 diabetes (T1D) is a chronic immune-mediated disease characterized by destruction of *β* cells, the insulin-producing cells in the pancreas. Trafficking of autoreactive T lymphocytes to the pancreatic islets and the resulting inflammatory environment culminates in disease establishment in at-risk individuals [1]. Approximately 19 million people have T1D worldwide, and this number has increased over the years [2]. After diagnosis, lifelong administration of exogenous insulin is required, and this is the only therapy currently available. Moreover, these individuals have to learn how to manage their condition and be aware of the possible disease-related complications.

To study the pathogenesis and immunophysiology of T1D, some murine models have been developed over the years, each one sharing different mechanisms of the human disease. Chemical induction of T1D through multiple doses of streptozotocin (STZ) is an immune-mediated T1D model discovered by Like and Rossini [3–5]. The STZ-induced model exhibits a phenotype and cellular infiltration similar to those observed in human disease, with a humoral response and autoreactive T cells against pancreatic antigens [6–8]. Another animal model, nonobese diabetic (NOD) mice are an inbred strain developed by Makino et al. from Jcl:ICR mice and one of the most commonly used models in T1D research [9, 10]. NOD mice spontaneously develop autoimmune diabetes and exhibit important features observed in human T1D, such as genetic susceptibility, diabetes-specific autoantibodies, and lymphocytic infiltration of pancreatic islets [11–13].

Cytokines play a key role in connecting the risk factors that contribute to T1D development and trigger the destructive action of T cells in pancreatic islets [14, 15]. In this context, studies investigating the potential of interleukin- (IL-) 4- and IL-10-mediated immunomodulation of T1D through individual or combined administration have shown that this is a new field to be explored [16–19]. In 2012, Mandke and Singh injected cationic nanomicelles intramuscularly to deliver a plasmid encoding IL-4 and IL-10 before STZ induction of T1D, resulting in protection against insulitis. However, entry of the delivery system into the muscle caused the infiltration of a large number of macrophages and lymphocytes at the site of injection [20].

The recurrent problem of biocompatibility and an undesirable immune response during the delivery process encouraged us to use *Lactococcus lactis* as the vehicle for mucosal delivery of plasmids encoding IL-4 and IL-10. This lactic acid bacteria also have been handled as a genetic tool due to some advantages, as do not colonize the tract gastrointestinal, present safe use in humans, and be able to induce tolerance successfully [21, 22]. *L. lactis* bacteria have been engineered to express a variety of molecules, including an invasin called fibronectin-binding protein A (FnBPA) (*L. lactis* MG1363 FnBPA^+^ strain) to more efficiently deliver the eukaryotic expression plasmids encoding IL-4 and IL-10 [23, 24]. Thus, administration of *L. lactis* MG1363 FnBPA^+^ (pValac::dts::*IL-4*) and *L. lactis* MG1363 FnBPA^+^ (pValac::*IL-10*) allows an increased rate of internalization and plasmid transference to eukaryotic cells, which will be able to express IL-4 and IL-10. Systematic administration of these cytokines was shown to be a successful approach in studies involving T1D [25–27]. Moreover, it has already been shown that immune responses triggered by intestinal stimuli can modulate the development of T1D, although the underlying mechanisms remain unclear [28, 29]. Thus, the oral delivery becomes an attractive route to administer the treatment, since the internalization of bacteria by mammalian host cells and subsequent expression of the plasmids are crucial steps in our strategy.

In the present study, we examined the effect of oral administration of *L. lactis* MG1363 FnBPA^+^ (pValac::dts::*IL-4*) and *L. lactis* MG1363 FnBPA^+^ (pValac::*IL-10*) in the two most extensively used animal models of T1D, the STZ-induced model, and NOD mice. The combined treatment of these bacterial strains for the first time is an interesting approach, since individual administration has already been shown to be able to reduce inflammation in intestinal inflammation models by our research group [30–32]. Therefore, we hypothesized that combined treatment with *L. lactis* MG1363 FnBPA^+^ strains carrying eukaryotic expression plasmids encoding IL-4 and IL-10 could result in beneficial effects on T1D.

## 2. Materials and Methods

### 2.1. Bacterial Strains and Growth Conditions

The strains used in this work are listed in Table 1. *L. lactis* MG1363 FnBPA^+^, *L. lactis* MG1363 FnBPA^+^ (pValac::dts::*IL-4*), and *L. lactis* MG1363 FnBPA^+^ (pValac::*IL-10*) were grown in M17 medium (Sigma-Aldrich) supplemented with 0.5% glucose with or without chloramphenicol (10 *μ*g/mL) (Sigma-Aldrich) and erythromycin (5 *μ*g/mL) (Sigma-Aldrich) at 30°C.

### 2.2. Animals

Male C57BL/6 mice, aged 8-9 weeks, were obtained from Biotério Central of Federal University of Minas Gerais (UFMG; Belo Horizonte, MG/Brazil). Female NOD mice were kindly donated by Dr. Ana Maria Caetano de Faria from UFMG. The animals were kept in microisolators on ventilated shelves with air filtration under a 12 h light-dark cycle with free access to standard mouse chow and water. All animal procedures were approved by the Ethics Committee on Animal Use (CEUA, Protocol no: 206/2016) of UFMG/Brazil.

### 2.3. Experimental Design and Diabetes Monitoring

**Figure 1:**
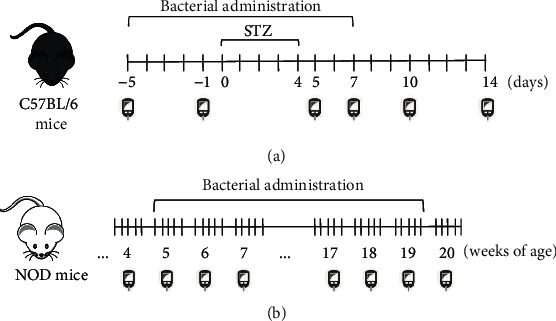
Experimental designs of the STZ-induced and NOD mouse models. (a) Diabetes was induced in male C57BL/6 mice by intraperitoneal injection of STZ + sodium citrate buffer for 5 consecutive days (days 0–4). Animals received bacterial administration daily from day -5 to day 7. Blood samples were collected on days -5, -1, 5, 7, 10, and 14 to measure glycemia. Animals were euthanized on day 14 of the experiment. (b) NOD mice received intragastric administration of bacterial suspensions 5 days/week for 15 consecutive weeks. Glycemic levels were measured weekly during the experiment. Animals were euthanized at the end of the 20th week.

C57BL/6 mice were subjected to a 4 h fast and were then injected intraperitoneally (i.p.) with 50 mg/kg/day STZ (Sigma-Aldrich) for 5 consecutive days (days 0-4) (Figure 1(a)). STZ was diluted in 0.1 M sodium citrate buffer, pH 4.5, and injected into the mice immediately after preparation. Subsequently, the animals were subjected to a 2 h fast. The vehicle control group received only sodium citrate buffer i.p. From day -5 until day 7, the animals were intragastrically (i.g.) administered 100 *μ*L of the corresponding *L. lactis* strain at a final dose of 1 × 10^9^ colony forming units (CFU) in 0.9% saline.

NOD mice were i.g. administered 100 *μ*L of bacterial suspension at a final dose of 1 × 10^9^ CFU in 0.9% saline 5 times/week for 15 consecutive weeks (Figure 1(b)).

Blood samples were collected from the tail vein of nonfasted mice on days -5, -1, 5, 7, 10, and 14 in the STZ-induced model (Figure 1(a)) and once a week during the experiments using the NOD mouse model (Figure 1(b)). Glucose levels were determined using a Accu-Chek Active glucometer system (Roche), and mice were considered diabetic when glucose levels were ≥200 mg/dL during two consecutive measurements.

### 2.4. Study Groups

C57BL/6 mice were allocated into groups that received 0.9% saline i.g., and by intraperitoneal route, the animals received 0.9% saline (negative control group, –) or sodium citrate buffer (vehicle control (VC) group) and groups that received the STZ injections i.p. and, by gavage, administration of 0.9% saline (saline-STZ group), *L. lactis* MG1363 FnBPA^+^ (F-STZ group), *L. lactis* MG1363 FnBPA^+^ (pValac::dts::*IL-4*) (F-IL-4-STZ group), *L. lactis* MG1363 FnBPA^+^ (pValac::*IL-10*) (F-IL-10-STZ group), *L. lactis* MG1363 FnBPA^+^ (pValac::dts::*IL-4*), and *L. lactis* MG1363 FnBPA^+^ (pValac::*IL-10*) (F-IL-4/IL-10-STZ group).

NOD mice were distributed into two groups, according to the constitution of dose i.g.: 0.9% saline (saline-NOD group) and *L. lactis* MG1363 FnBPA^+^ (pValac::dts::*IL-4*), and *L. lactis* MG1363 FnBPA^+^ (pValac::*IL-10*) (F-IL-4/IL-10-NOD group).

### 2.5. Cytokine Detection

The blood, the pancreas, and the colon were harvested immediately after euthanasia. Blood samples were incubated at 37°C for 40 minutes and then centrifuged at 5,000 rpm for 10 minutes at 4°C. The serum was collected and stored at -20°C until use. Pancreatic and colon tissues were homogenized in 1 mL buffer (23.4 g NaCl; 500 *μ*L Tween 20; 5 g BSA; 34 mg PMSF; 1 mL DMSO; 44.6 mg BC; 372 mg Na_2_EDTA; and 40 *μ*L aprotinin (10 mg/mL) (Sigma-Aldrich); PBS 1× q.s.p. 1 L) per 100 mg of tissue. Then, the samples were centrifuged at 10,000 rpm for 10 minutes at 4°C, and the supernatant was stored at -80°C until use.

The concentrations of IL-4 and IL-10 were measured in serum, and the levels of IL-2, IL-4, IL-10, IL-12, and transforming growth factor *β* (TGF-*β*) in pancreatic and colon homogenates were determined using BD OptEIA (BD) and R&D DuoSet (R&D Systems) ELISA kits, according to the manufacturers' instructions.

### 2.6. Secretory IgA Detection

The concentration of secretory immunoglobulin A (sIgA) in intestinal lavage fluid was measured by capture ELISA using goat anti-mouse Ig, human ads-UNLB (Southern Biotech), and goat anti-mouse IgA (*α* chain specific) horseradish peroxidase (HRP) conjugate (Southern Biotech), as recommended by the manufacturer.

### 2.7. Bacterial Translocation

C57BL/6 mice received 100 *μ*L of bacterial suspension (*L. lactis* MG1363 FnBPA^+^ (pValac::dts::*IL-4*) and *L. lactis* MG1363 FnBPA^+^ (pValac::*IL-10*)) at a final dose of 1 × 10^9^ CFU in 0.9% saline by oral gavage. The spleen, liver, and mesenteric lymph nodes (MLNs) were collected under strict aseptic conditions 2 h, 4 h, 6 h, 12 h, and 24 h after bacterial administration. The tissues were macerated in 500 *μ*L of 0.9% saline, and 100 *μ*L of this homogenate was plated in M17 medium (Sigma-Aldrich) supplemented with agar, 0.5% glucose, chloramphenicol (10 *μ*g/mL) (Sigma-Aldrich), and erythromycin (5 *μ*g/mL) (Sigma-Aldrich). The plates were incubated at 30°C for 24 h.

### 2.8. Histological Analysis

After euthanasia, the pancreas was removed, fixed in 10% buffered formalin, embedded in paraffin, and sectioned. Five-*μ*m sections were stained with hematoxylin and eosin (HE), and at least 15 islets per pancreatic sample were evaluated and scored for infiltration progression (by an independent investigator in blinded fashion), as follows: no insulitis: no visible signs of infiltration; peri-insulitis: few infiltrating elements with patchy distribution primarily at the periphery of the islet; mild insulitis: mild infiltration (peri- and intraislet) with less than 50% of the islet infiltrated by the immune elements; and severe insulitis: extensive inflammation with more than 50% of the islet infiltrated.

### 2.9. Statistical Analysis

The GraphPad Prism software, version 5.0 (GraphPad), was used for statistical analysis, and the results are presented as the mean ± standard deviation (SD). One-way ANOVA followed by Tukey's post hoc test was used for the analysis of variance. For diabetes incidence, the *χ*^2^ test was used. *p* values less than 0.05 (*p* < 0.05) were considered statistically significant.

## 3. Results

### 3.1. Effect of Genetically Modified *L. lactis* on Clinical Manifestations in T1D Models

**Figure 2:**
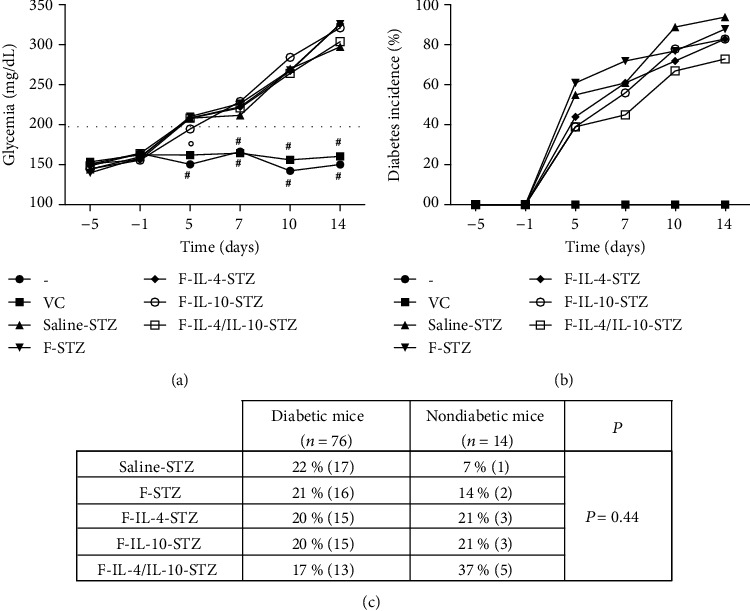
Clinical effects of genetically modified *L. lactis* strains encoding IL-4 and IL-10 in the STZ-induced diabetes model. C57BL/6 mice were injected with 0.9% saline, vehicle solution, or STZ for five consecutive days (50 mg/kg/d). Specific bacterial administration was performed daily by the intragastric route from day -5 to day 7. (a) Glycemic levels (mg/dL) were measured at days -5, -1, 5, 7, 10, and 14. The data are shown as the mean ± SD of three independent experiments (*n* = 18). #: experimental group whose glycemia was significantly different from the saline-STZ, F-STZ, F-IL-4-STZ, F-IL-10-STZ, and F-IL-4/IL-10-STZ groups; °: experimental group whose glycemia was significantly different from the saline-STZ, F-STZ, F-IL-4-STZ, and F-IL-4/IL-10-STZ groups. (b) Diabetes incidence over time was analyzed, and (c) the percentage of diabetic and nondiabetic mice between the experimental groups and the disease control group at day 14 was compared. *p* value: *p* ≤ 0.05.

Glycemic levels were analyzed, and oral bacterial treatment of STZ-induced groups (F-STZ, F-IL-4-STZ, F-IL-10-STZ, and F-IL-4/IL-10-STZ groups) did not alter the increases in glycemic levels at any time after T1D induction; blood glucose levels in these groups were similar to that in the saline-STZ group (Figure 2(a)). A progressive increase in diabetes incidence was also observed in groups that received STZ injections, regardless of oral treatment administration (Figure 2(b)).  Figure 2(c) shows the comparison of the percentage of diabetic and nondiabetic mice between the experimental groups (F-STZ, F-IL-4-STZ, F-IL-10-STZ, and F-IL-4/IL-10-STZ groups) and the disease control group (saline-STZ group) at the final time point. The distribution of diabetic mice was analyzed, and by day 14, all groups showed similar percentages. However, the F-IL-4/IL-10-STZ group exhibited the highest percentage of nondiabetic mice (37%).

**Figure 3:**
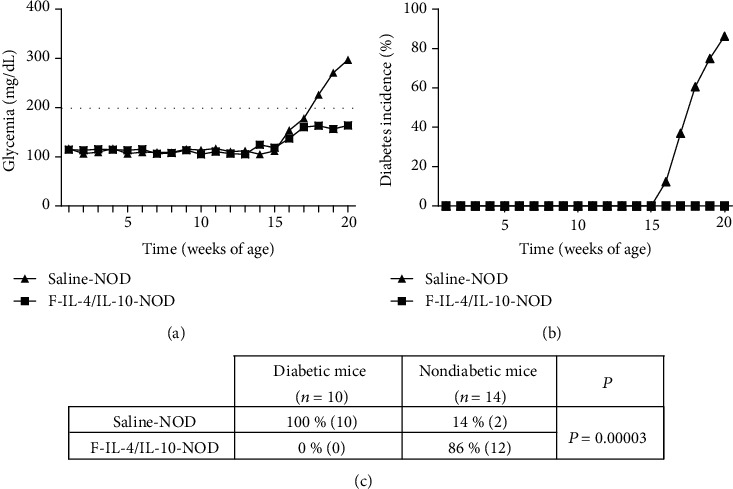
Clinical effects of genetically modified *L. lactis* strains encoding IL-4 and IL-10 in the NOD mouse model. NOD mice were i.g. administered *L. lactis* MG1363 FnBPA^+^ (pValac::dts::*IL-4*) and *L. lactis* MG1363 FnBPA^+^ (pValac::*IL-10*) suspension (F-IL-4/IL-10-NOD group) or 0.9% saline (saline-NOD group) 5 days/week from week 5 to week 19. (a) Glycemic levels (mg/dL) were measured once a week from weeks 1 to 20. The data are shown as the mean ± SD of three independent experiments (*n* = 12). (b) Diabetes incidence over time was analyzed, and (c) the percentage of diabetic and nondiabetic mice between the NOD mouse groups at the final time point was compared. *p* value: *p* ≤ 0.05.

In contrast to the results observed in the STZ model, NOD mice that received oral treatment with *L. lactis* MG1363 FnBPA^+^ (pValac::dts::*IL-4*) and *L. lactis* MG1363 FnBPA^+^ (pValac::*IL-10*) (F-IL-4/IL-10-NOD group) showed normoglycemia (glycemic levels less than 200 mg/dL) until the end of the experimental period (Figure 3(a)) and treatment protected NOD mice from T1D development. Analysis showed that diabetes incidence increased in the saline-NOD group from week 15 onwards, while mice in the F-IL-4/IL-10-NOD group remained protected against diabetes during the entire experimental period (Figure 3(b)). The percentages of diabetic and nondiabetic mice per group, among all NOD mice, were statistically significant, as shown in Figure 3(c). By 20th week, all diabetic mice belonged to the saline-NOD group, and there were no diabetic mice in the F-IL-4/IL-10-NOD group. In addition, analysis of the nondiabetic mice showed that only 14% of animals were in the saline-NOD group, while the remaining 86% were in the F-IL-4/IL-10-NOD group. The comparison of glycemia and the percentage of diabetes incidence in groups of STZ-induced mice and NOD mice at the final time point (Table 2) corroborates the previous analysis.

### 3.2. Cytokine Levels in the Serum and Pancreas Are Differentially Modulated in STZ-Induced and NOD Mouse Models of Diabetes

**Figure 4:**
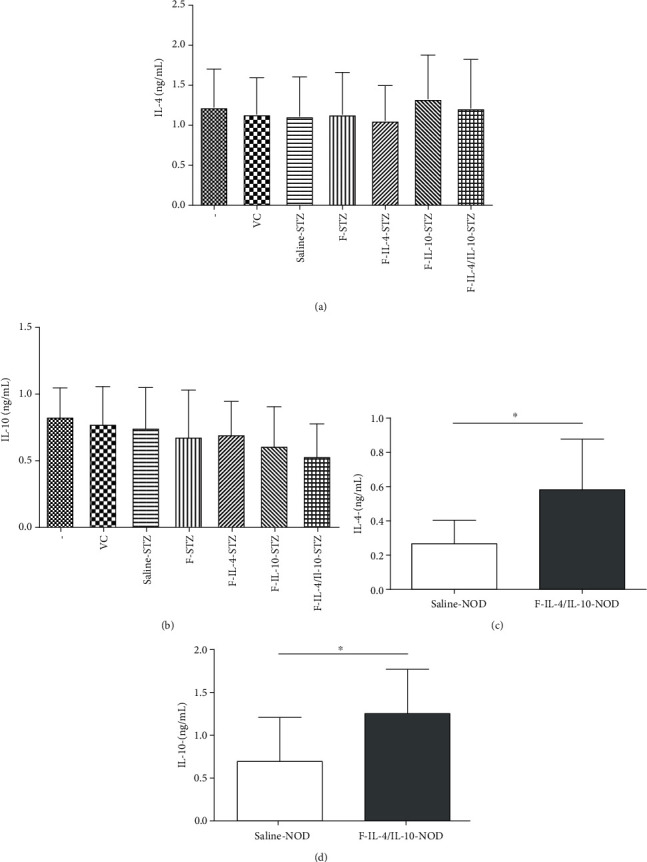
Serum levels of IL-4 and IL-10 from STZ-induced C57BL/6 mice and NOD mice, which were both treated with oral bacterial administration. Saline, vehicle, or STZ-injected C57BL/6 mice were euthanized at the end of the experiment, and serum was collected to measure IL-4 and IL-10 levels ((a, b), respectively, *n* = 18). After saline or oral bacterial administration, serum was collected from NOD mice after euthanasia to measure IL-4 and IL-10 levels ((c, d), respectively, *n* = 12). –: negative control; VC: vehicle control; F: mice that received *L. lactis* MG1363 FnBPA^+^ i.g.; F-IL-4: mice that received *L. lactis* MG1363 FnBPA^+^ (pValac::dts::*IL-4*) i.g.; F-IL-10: mice that received *L. lactis* MG1363 FnBPA^+^ (pValac::*IL-10*) i.g.; F-IL-4/IL-10: mice that received *L. lactis* MG1363 FnBPA^+^ (pValac::dts::*IL-4*) and *L. lactis* MG1363 FnBPA^+^ (pValac::*IL-10*) i.g.; STZ: mice that received intraperitoneal injections of streptozotocin for five consecutive days; NOD: nonobese diabetic mice. The data are shown as the mean ± SD of three independent experiments. *p* value: ^∗^*p* ≤ 0.05.

Cytokine profiles in serum and the pancreas were investigated in both T1D models, and in the STZ-induced model, all groups presented similar serum levels of IL-4 and IL-10 (Figures 4(a) and 4(b), respectively). In contrast, NOD mice treated with *L. lactis* MG1363 FnBPA^+^ (pValac::dts::*IL-4*) and *L. lactis* MG1363 FnBPA^+^ (pValac::*IL-10*) exhibited significantly higher serum production of IL-4 (Figure 4(c)) and IL-10 (Figure 4(d)) than the saline-NOD group.

**Figure 5:**
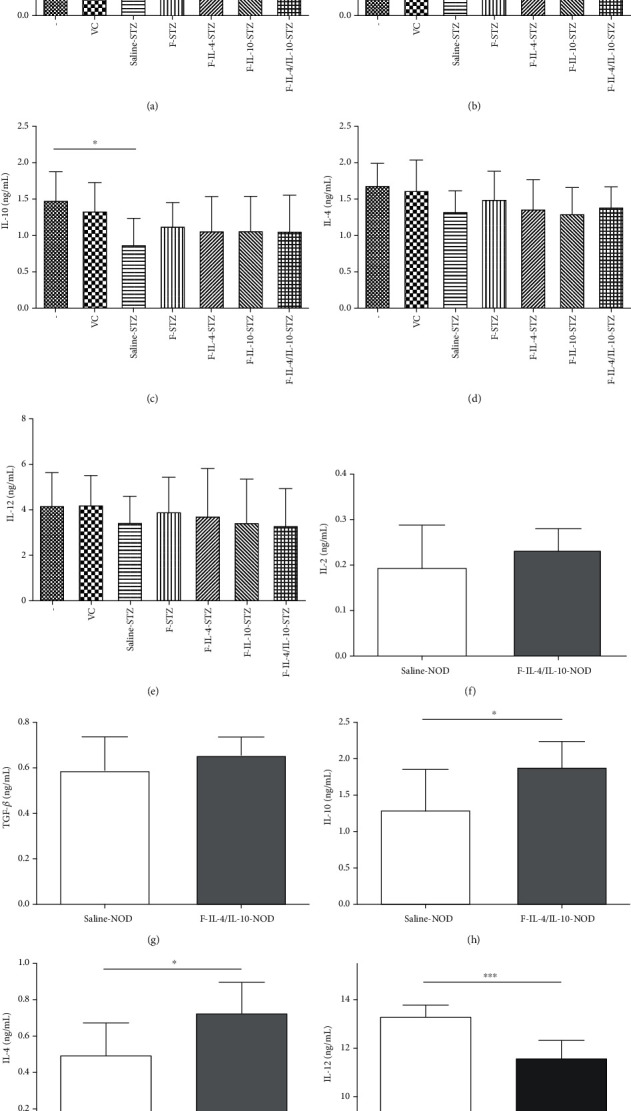
Pancreatic cytokine levels of STZ-induced C57BL/6 mice and NOD mice, which were both treated with oral bacterial administration. After the experimental period, the levels of IL-2, TGF-*β*, IL-10, IL-4, and IL-12 were analyzed in pancreatic homogenates from STZ model mice ((a–e), respectively; *n* = 18) and NOD mice ((f–j), respectively; *n* = 12). –: negative control; VC: vehicle control; F: mice that received *L. lactis* MG1363 FnBPA^+^ i.g.; F-IL-4: mice that received *L. lactis* MG1363 FnBPA^+^ (pValac::dts::*IL-4*) i.g.; F-IL-10: mice that received *L. lactis* MG1363 FnBPA^+^ (pValac::*IL-10*) i.g.; F-IL-4/IL-10: mice that received *L. lactis* MG1363 FnBPA^+^ (pValac::dts::*IL-4*) and *L. lactis* MG1363 FnBPA^+^ (pValac::*IL-10*) i.g.; STZ: mice that received intraperitoneal injections of streptozotocin for five consecutive days; NOD: nonobese diabetic mice. The data are shown as the mean ± SD of three independent experiments. *p* value: ^∗^*p* ≤ 0.05; ^∗∗^*p* ≤ 0.01; ^∗∗∗^*p* ≤ 0.001.

Pancreatic levels of IL-2 were analyzed, and other than the negative and vehicle controls in the STZ model, F-IL-4/IL-10-STZ was the only group that exhibited significantly higher IL-2 levels than the saline-STZ group (Figure 5(a)). Regarding TGF-*β* and IL-10 levels, a significant difference was found only between the negative and saline-STZ control groups (Figures 5(b) and 5(c), respectively). IL-4 (Figure 5(d)) and IL-12 (Figure 5(e)) levels were similar among all pancreatic samples.

The levels of pancreatic cytokines in NOD mice were measured, and we found that IL-2 (Figure 5(f)) and TGF-*β* (Figure 5(g)) levels were similar in both groups. On the other hand, IL-10 production was significantly higher in NOD mice that received *L. lactis* MG1363 FnBPA^+^ (pValac::dts::*IL-4*) and *L. lactis* MG1363 FnBPA^+^ (pValac::*IL-10*) than in the saline-NOD group (Figure 5(h)). Furthermore, the F-IL-4/IL-10-NOD group also had significantly increased production of IL-4 in comparison to that in the saline-NOD group (Figure 5(i)). And the level of pancreatic IL-12 was significantly lower in NOD mice treated with bacterial strains (F-IL-4/IL-10-NOD group) than in control mice (Figure 5(j)).

### 3.3. Changes in sIgA Levels but Not Cytokine Production Were Detected in the Intestinal Colon Environment

**Figure 6:**
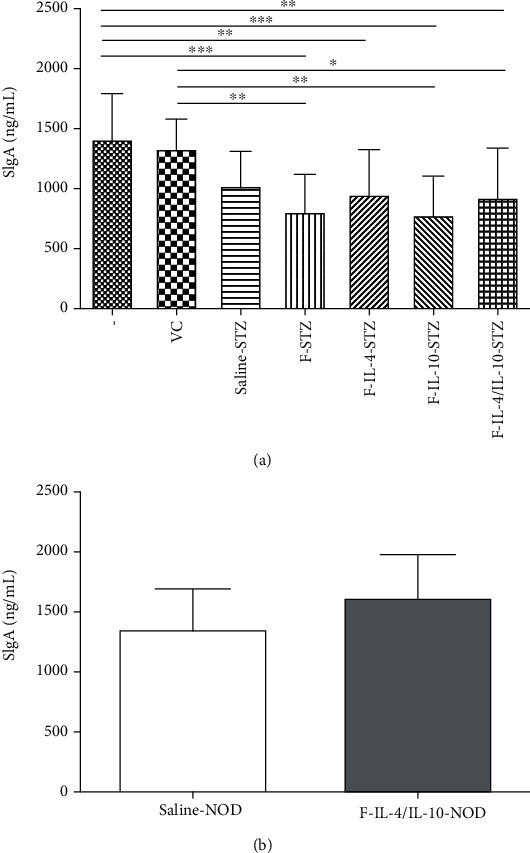
sIgA levels in intestinal lavage fluid from STZ-induced C57BL/6 mice and NOD mice, which were both treated with oral bacterial administration. Secretory IgA levels from (a) STZ-induced C57BL/6 mice (*n* = 18) and (b) NOD mice (*n* = 12). –: negative control; VC: vehicle control; F: mice that received *L. lactis* MG1363 FnBPA^+^ i.g.; F-IL-4: mice that received *L. lactis* MG1363 FnBPA^+^ (pValac::dts::*IL-4*) i.g.; F-IL-10: mice that received *L. lactis* MG1363 FnBPA^+^ (pValac::*IL-10*) i.g.; F-IL-4/IL-10: mice that received *L. lactis* MG1363 FnBPA^+^ (pValac::dts::*IL-4*) and *L. lactis* MG1363 FnBPA^+^ (pValac::*IL-10*) i.g.; STZ: mice that received intraperitoneal injections of streptozotocin for five consecutive days; NOD: nonobese diabetic mice. The data are shown as the mean ± SD of three independent experiments. *p* value: ^∗^*p* ≤ 0.05; ^∗∗^*p* ≤ 0.01; ^∗∗∗^*p* ≤ 0.001.

The levels of several cytokines (IL-2, IL-4, IL-10, IL-12, and TGF-*β*) in the colon homogenates from STZ-induced and NOD mouse models were measured and did not show significant differences among the groups (data not shown). However, there was decreased production of sIgA in all C57BL/6 mice that received bacterial strains (F-STZ, F-IL-4-STZ, F-IL-10-STZ, and F-IL-4/IL-10-STZ groups) in comparison to that of groups that received only oral saline (negative and VC groups) (Figure 6(a)). Interestingly, as shown in Figure 6(b), the saline-NOD and F-IL-4/IL-10-NOD groups exhibited similar levels of sIgA.

### 3.4. Detection of Orally Administered Bacterial Strains in the Liver and Mesenteric Lymph Nodes Was Transient

**Figure 7:**
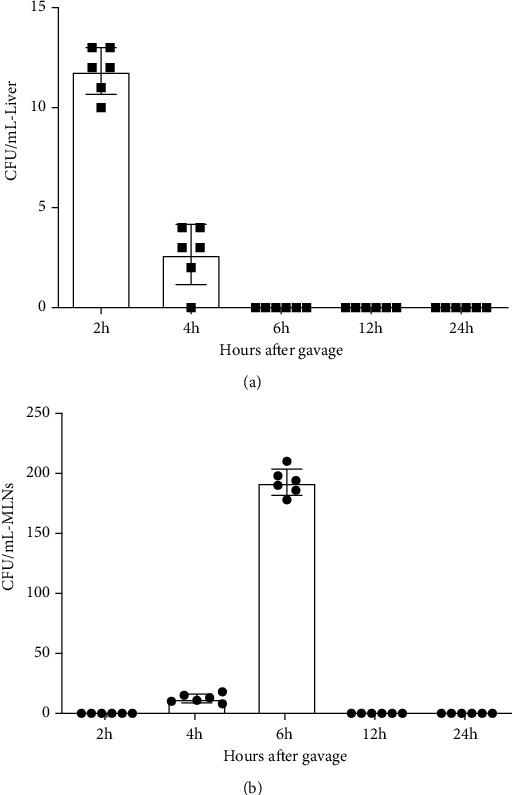
Transient translocation of *L. lactis* MG1363 FnBPA^+^ (pValac::dts::*IL-4*) and *L. lactis* MG1363 FnBPA^+^ (pValac::*IL-10*) was detected in the liver and MLNs. The organs were collected 2 h, 4 h, 6 h, 12 h, and 24 h after bacterial administration, macerated in 0.9% saline, and plated on selective medium. After 24 h of incubation at 30°C, the bacterial colonies in the plates corresponding to the liver (a) and MLNs (b) were counted. All results are presented as colony forming units (CFU) per mL. The data are shown as the mean of three independent experiments (*n* = 6).

Translocation of the orally administered bacterial strains (*L. lactis* MG1363 FnBPA^+^ (pValac::dts::*IL-4*) and *L. lactis* MG1363 FnBPA^+^ (pValac::*IL-10*)) was not detected in the spleen (data not shown) at 24 h after gavage. However, we observed a minute quantity of bacterial translocation in the liver at 2 h and 4 h after oral administration of the bacteria (Figure 7(a)). Additionally, we also verified the presence of *L. lactis* MG1363 FnBPA^+^ (pValac::dts::*IL-4*) and *L. lactis* MG1363 FnBPA^+^ (pValac::*IL-10*) in MLNs, primarily at 6 h after oral gavage (Figure 7(b)). At 12 h and 24 h after bacterial administration, none of the genetically modified strains of *L. lactis* were found in the liver or MLNs.

### 3.5. Insulitis Progression Was Prevented by Combined Treatment with *L. lactis* Carrying Plasmids Encoding IL-4 and IL-10

**Figure 8:**
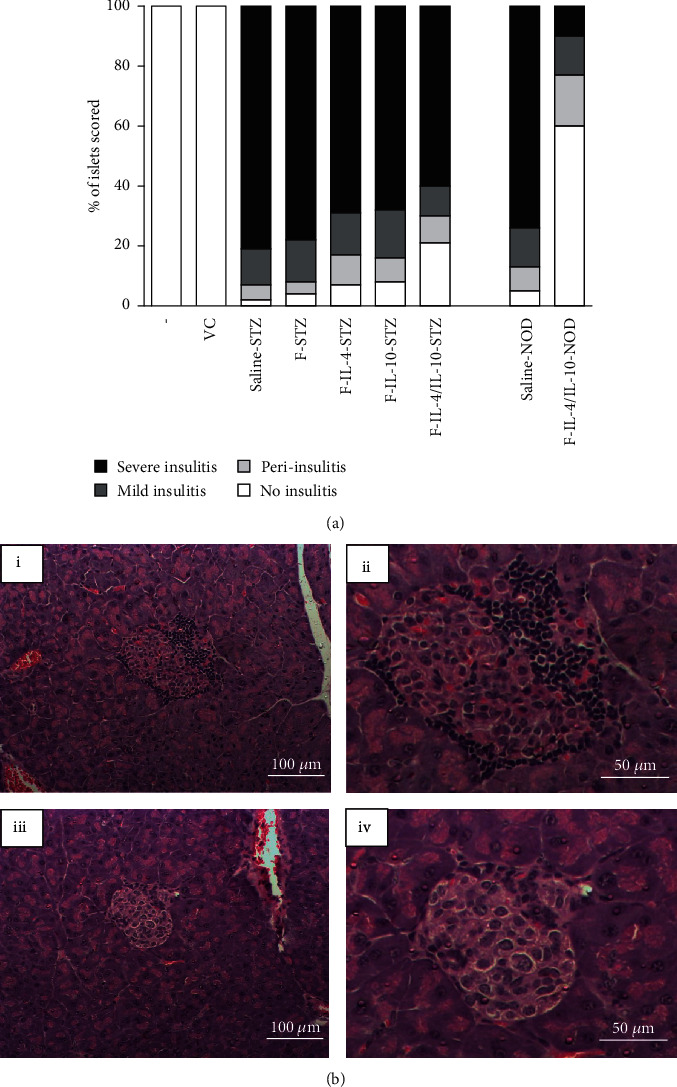
Combined administration of *L. lactis* MG1363 FnBPA^+^ (pValac::dts::*IL-4*) and *L. lactis* MG1363 FnBPA^+^ (pValac::*IL-10*) exerts protective effects on pancreatic islets. (a) Insulitis scoring was performed in a blind manner on paraffin-embedded pancreatic sections of STZ-induced mice and NOD mice at the end of experimental period. At least 15 islets were evaluated in each pancreatic sample. The data are shown as the mean of three independent experiments (*n* = 6). (b) A representative pancreatic islet from the saline-NOD group (i-ii) and F-IL-4/IL-10-NOD group (iii-iv) is shown.

Histological analysis demonstrated that treatment with both bacterial strains (*L. lactis* MG1363 FnBPA^+^ (pValac::dts::*IL-4*) and *L. lactis* MG1363 FnBPA^+^ (pValac::*IL-10*); (F-IL-4/IL-10-STZ group)) in STZ-induced mouse model preserved more pancreatic islets without visible signs of inflammation than when these strains were administered individually (F-IL-4-STZ and F-IL-10-STZ groups) (Figure 8(a)). As shown in Figures 8(a) and 8(b), insulitis scores also revealed that NOD mice treated with the combined administration of the bacterial strains encoding IL-4 and IL-10 successfully prevented inflammation progression in pancreatic islets during the observational period. Analysis demonstrated that mice from the F-IL-4/IL-10-NOD group presented 60% of the islets without signs of insulitis, compared with 5% in the saline-NOD control group.

## 4. Discussion

T1D is a chronic condition in which pancreatic *β* cells are destroyed by autoreactive T cells. After diagnosis, patients have to adapt their lives in an attempt to reach wellness mainly because the available treatment, insulin therapy, is laborious and requires constant care and attention. Therefore, more studies investigating T1D development and improved treatments are absolutely needed.

Early studies using multiple doses of STZ and NOD mice have shown that IL-4 and IL-10 have the potential to change the natural course of T1D by impairing insulitis and maintaining normal levels of glycemia [20, 34, 35]. However, the route of administration and delivery strategy of these cytokines in animal models of T1D is a concerning challenge. Thus, we decided to use the recombinant *L. lactis*, since it is considered an ideal tool to mucosal delivery and oral tolerance induction [22]. Oral administration of *L. lactis* MG1363 FnBPA^+^ strains carrying eukaryotic expression vectors encoding IL-4 and IL-10 (pValac::dts::*IL-4* and pValac::*IL-10*, respectively) to treat the two most extensively used animal models of T1D is reported here for the first time.

In the STZ-induced T1D model, we decided to verify the effects of the bacterial strains carrying the plasmids encoding IL-4 and IL-10 individually and combined. Despite all groups that received STZ injections became diabetic, it was noteworthy that the F-IL-4/IL-10-STZ group presented the highest number of mice protected against diabetes incidence.

Moreover, STZ-injected mice orally administered both bacterial strains encoding IL-4 and IL-10 showed pancreatic IL-2 levels similar to those of the negative and vehicle control groups and were significantly higher than the saline-STZ group. Previous studies reported that IL-2 plays a key role in the balance between immunoregulation and immunosuppression, since this cytokine is able to expand and activate regulatory T cells (Tregs), which subsequently provide active suppression of the effector response [36, 37]. Particularly in T1D, low doses of IL-2 can prevent the development of the disease [38]. It has already been reported that Tregs from patients with diabetes exhibit deficient IL-2 production and/or signaling, and if the IL-2 pathway is blocked, the frequency of Tregs decreases, and the number of individuals affected by T1D increases [39]. Thus, the normal levels of IL-2 in STZ-injected mice treated with both bacterial strains carrying plasmids encoding IL-4 and IL-10 may have contributed to Treg activation in the pancreas and attenuated insulitis progression.

The evaluation of sIgA levels is important because it is already well established that this antibody exhibits immunomodulatory properties and is involved in not only responding to pathogens but also maintaining homeostasis between the gastrointestinal tract and commensal and ingested microorganisms [40–42]. In addition, low sIgA production may be related to a malfunction in immune cells in Peyer's patches in some immune-mediated diseases, such as celiac disease and T1D [43]. In the STZ-induced model, we noted that groups treated with the bacteria during few days had significantly reduced sIgA levels in the gut. However, the NOD mice that received bacteria orally for 15 weeks presented sIgA levels similar to those mice that did not receive bacteria. We believe this difference in sIgA levels between the T1D models can be explained by the long-term ingestion of bacteria in the NOD mouse model, which may have induced oral tolerance in mice, as previously reported by other studies using recombinant *L. lactis* by oral route [22].

Corroborating the others analysis, histological findings confirmed that combined treatment with the bacterial strains encoding IL-4 and IL-10 was more successful in attenuate insulitis progression than the individual administration of these bacterial strains. Results indicating more pancreatic islets with no signs of inflammatory infiltrates suggest a beneficial effect resulting from the combined bacterial strains. Some studies have shown that IL-4 and IL-10 induce a synergistic effect to suppress cellular immunity [44, 45]. Although the underlying mechanism is not well known, it is suggested that the combined and distinct actions of these cytokines could contribute to an enhanced outcome [26]. Since this is the first study using genetically modified *L. lactis* in the STZ-induced T1D model, these results were important and led to the investigation of the effects of the combined administration of *L. lactis* MG1363 FnBPA^+^ strains carrying the IL-4 and IL-10-coding plasmids in the NOD mouse model.

It is commonly known that the high and uncontrolled levels of glycemia are associated with a rapid progression of disease and more severe complications in patients with T1D. Using the NOD mouse model, we demonstrated that oral administered *L. lactis* MG1363 FnBPA^+^ (pValac::dts::*IL-4*) and *L. lactis* MG1363 FnBPA^+^ (pValac::*IL-10*) are able to regulate glycemia levels effectively throughout the observational period. Therefore, the oral treatment with these genetically modified bacteria during 15 weeks resulted in the absence of T1D incidence in NOD mice.

In the present study, we demonstrated that oral administration of the bacterial strains led to systemic effects. NOD mice treated with *L. lactis* MG1363 FnBPA^+^ encoding IL-4 and IL-10 by oral route presented significantly increased levels of IL-10 in serum and pancreatic tissue. IL-10 is a major anti-inflammatory cytokine that can modulate immune processes, inhibiting the synthesis of proinflammatory cytokines and favoring a tolerant status [46]. IL-10 has been shown to be active at mucosal surfaces, acting synergistically and enhancing oral tolerance in NOD mouse model [47]. IL-10 can also stabilize Treg function through maintenance of the Foxp3 expression [48]. Moreover, Takiishi et al. suggest that the production of IL-10 by Treg cells is the major mechanism responsible by regulation of inflammation reached by *L. lactis-*based therapy in the NOD mouse model [49]. T1D is related to inflammation, and hence, it is reasonable to speculate that increased levels of IL-10 obtained in serum and pancreas of NOD mice treated with the genetically modified *L. lactis* encoding IL-4 and IL-10 was at least initially due to the bacteria administration and subsequent expression of these cytokines. We suggest this result was crucial for regulation of inflammation observed, probably through Treg cells, and consequent control of glycemia and no incidence of T1D.

IL-4 is a pleiotropic immunomodulatory cytokine produced and secreted primarily by Th2 cells. Approaches favoring Th2 activity and administration of cytokines that downregulate Th1 activities, such as IL-4 and IL-10, have shown success in suppress insulitis and prevent diabetes [17, 18, 26]. Previous studies showed that both administration of recombinant IL-4 and pancreatic expression of IL-4 were able to protect NOD mice from diabetes [17, 50]. Furthermore, Li et al. also revealed that alteration in cytokine production in T cells, skewing to IL-4 production may contribute to disease protection in NOD mice [51]. In agreement with previously reported results, oral treatment with *L. lactis* MG1363 FnBPA^+^ (pValac::dts::*IL-4*) and *L. lactis* MG1363 FnBPA^+^ (pValac::*IL-10*) showed promising results indicating increased levels of IL-4 in serum and pancreas of NOD mice, further corroborating the beneficial role of the strategy tested here for the prevention of T1D.

Additionally, both IL-4 and IL-10 appear to suppress the production of the proinflammatory cytokine IL-12 by antigen-presenting cells (APCs), thereby downregulating the differentiation of Th1 cells [52, 53]. Accumulating evidence indicates that IL-12 is a key cytokine which induces the differentiation of Th1 cells leading to diabetes acceleration in NOD mice [54–56]. Additionally, Nitta et al. reported that IL-12 locally produced by pancreatic-infiltrating cells plays a pathologic role in the development of T1D [57]. Therefore, we suggest the significant reduction observed in the pancreatic concentration of IL-12 of NOD mice treated with both bacterial strains contributed to the regulation of inflammation, not favoring the differentiation of Th1 cells nor the production of proinflammatory cytokines.

More clear evidence of the protective effect of the combined administration of *L. lactis* MG1363 FnBPA^+^ (pValac::dts::*IL-4*) and *L. lactis* MG1363 FnBPA^+^ (pValac::*IL-10*) was obtained by the histological analysis. Corroborating the normoglycemia presented by NOD mice that received oral treatment with both bacterial strains, the majority of pancreatic islets was preserved, without signs of insulitis. Collectively, our data indicate the preservation of pancreatic islets is a result of the reduced autoimmune destruction, triggered by the increased levels of IL-4 and IL-10 and the decreased levels of IL-12. Consequently, this alteration in cytokine production in pancreatic environment possibly altered the T cells subsets, skewing to Treg cell proliferation, which may have contributed to the disease protection.

Genetically modified *L. lactis* have been used as a versatile tool for immunotherapy in several inflammatory conditions, such as T1D [21]. Since these bacterial strains can survive the entire gastrointestinal tract and are used for delivery of plasmids and proteins to mucosal cells, we assessed bacterial translocation to some extragastrointestinal organs. Previous studies have shown that bacterial translocation can occur on a frequent basis even in healthy conditions [58–60]. Genetically modified *L. lactis* encoding IL-4 and IL-10 were detected mostly in MLNs, which are known to be the key site for oral tolerance. The induced Treg cells proliferate in MLNs and are subsequently released in circulation to modulate pancreatic beta cell autoimmune destruction in a IL-10-dependent manner [21, 61, 62]. Bresson et al. demonstrated the expansion of Treg cells producing IL-4 and IL-10 was determinant to suppress autoreactive response in animal models of T1D [63]. Promising studies using genetically modified *L. lactis* as a delivery tool of antigens and bioactive compounds revealed the induction of oral tolerance and proliferation of Treg cells were able to revert new-onset diabetes in NOD mice [49, 64]. Thus, we hypothesized the oral treatment with *L. lactis* MG1363 FnBPA^+^ (pValac::dts::*IL-4*) and *L. lactis* MG1363 FnBPA^+^ (pValac::*IL-10*) was able to protect against beta-cell destruction due to expansion of Treg cells producing IL-4 and IL-10 in pancreas. Additionally, neither genetically modified bacteria was detected in the MLNs, spleen, and liver from 12 h after oral administration, indicating that these strains are indeed transient, which is a relevant attribute in the context of biosafety practices.

Altogether, the protective effect against T1D triggered by the oral administration of *L. lactis* MG1363 FnBPA^+^ strains carrying pValac::dts::*IL-4* and pValac::*IL-10* verified in NOD mice was not observed in the STZ-induced model. This difference between the two main T1D models has already been reported in other studies [65–67], and it may be associated with the distinct characteristics of these models. The STZ model is characterized by rapid destruction of insulin-producing cells in the pancreas through a process involving dynamic variation of immune cells [68–70]. Since STZ is able to induce diabetes even in the absence of B and T cells, interpretation of the results requires prudence when the analysis is correlated with human development of T1D [71, 72]. In this context, NOD mice integrate the genetic component and the implications of the immunopathological process, which makes this a suitable model for testing therapies that aim to modulate the autoimmune response [72, 73].

Regarding the limitations of our study, we could not provide all the desirable experimental control groups with NOD mice, since the acquisition and maintenance of these animals are costly. However, the assessments performed with all groups in the STZ-induced model showed that we should focus efforts on the combined administration of bacterial strains carrying the cytokine-coding plasmids. Additionally, these limitations do not invalidate our valuable findings on combined administration of *L. lactis* MG1363 FnBPA^+^ strains carrying the eukaryotic expression plasmids encoding IL-4 and IL-10 in the NOD mouse model.

In conclusion, the results of our study demonstrate that the combined administration of *L. lactis* MG1363 FnBPA^+^ strains carrying the eukaryotic expression plasmids pValac::dts::*IL-4* and pValac::*IL-10* attenuated the initial progression of insulitis in the STZ-induced model by increasing levels of IL-2 in pancreatic tissue and protected NOD mice against T1D in the observational period. Our findings suggest the dynamic cytokine signaling triggered by the oral treatment with the genetically modified bacteria plays a crucial role in the immunomodulation of the disease in the NOD mouse model. Our results showed the oral treatment with *L. lactis* MG1363 FnBPA^+^ strains encoding IL-4 and IL-10 can effectively reduce the incidence of T1D and control the glycemia levels in NOD mice. Additionally, we demonstrated this combined treatment was able to prevent insulitis progression, primarily by increasing the levels of IL-4 and IL-10, known by suppressing the proinflammatory cytokine milieu, in pancreas and serum and decreasing the levels of the proinflammatory cytokine IL-12 in pancreas. This is the first study to administer orally the combined genetically modified *L. lactis* strains carrying eukaryotic expression plasmids encoding IL-4 and IL-10 in the STZ-induced and in the NOD mouse models. In summary, our study preliminarily provides novel and relevant observations, enhancing the potential of this innovative approach as part of an alternative therapy to T1D.

## Figures and Tables

**Table 1:** Bacterial strains used in this work.

Strain	Characteristics	Source
*Lactococcus lactis* MG1363 FnBPA^+^	*L. lactis* MG1363 strain expressing *S. aureus* FnBPA	Que et al. [33]
*Lactococcus lactis* MG1363 FnBPA^+^ (pValac::dts::*IL-4*)	*L. lactis* MG1363 FnBPA^+^ strain carrying the pValac::dts::*IL-4* plasmid	Souza et al. [32]
*Lactococcus lactis* MG1363 FnBPA^+^ (pValac::*IL-10*)	*L. lactis* MG1363 FnBPA^+^ strain carrying the pValac::*IL-10* plasmid	Zurita-Turk et al. [31]

FnBPA: fibronectin-binding protein A; IL-4: interleukin 4; IL-10: interleukin 10; dts: DNA nuclear targeting sequence.

**Table 2:** Comparison of clinical parameters observed in STZ model mice and NOD mice.

	STZ model: day 14							NOD mice: week 20	
	—	VC	Saline-STZ	F-STZ	F-IL-4-STZ	F-IL-10-STZ	F-IL-4/IL-10-STZ	Saline-NOD	F-IL-4/IL-10-NOD
Glycemia (mg/dL)	150	160	297	326	325	321	303	298	177
Diabetes incidence (%)	0	0	95	89	84	84	72	84	0

## Data Availability

The data used to support the findings of this study are included within the article.
